# Acceptability and feasibility of potential intervention strategies for influencing sedentary time at work: focus group interviews in executives and employees

**DOI:** 10.1186/s12966-015-0177-5

**Published:** 2015-02-18

**Authors:** Katrien De Cocker, Charlene Veldeman, Dirk De Bacquer, Lutgart Braeckman, Neville Owen, Greet Cardon, Ilse De Bourdeaudhuij

**Affiliations:** Department of Movement and Sport Sciences, Ghent University, Watersportlaan 2, B-9000 Ghent, Belgium; Research Foundation Flanders, Egmontstraat 5, B-1000 Brussels, Belgium; Department of Public Health, Ghent University, De Pintelaan 185, B-9000 Ghent, Belgium; Baker IDI Heart and Diabetes Institute, Level 4, 99 Commercial Rd, Melbourne, VIC 3004 Australia

**Keywords:** Occupational sitting, Worksite, Interviews, Reducing and interrupting sedentary time

## Abstract

**Background:**

Occupational sitting can be the largest contributor to overall daily sitting time in white-collar workers. With adverse health effects in adults, intervention strategies to influence sedentary time on a working day are needed. Therefore, the present aim was to examine employees’ and executives’ reflections on occupational sitting and to examine the potential acceptability and feasibility of intervention strategies to reduce and interrupt sedentary time on a working day.

**Methods:**

Seven focus groups (four among employees, n = 34; three among executives, n = 21) were conducted in a convenience sample of three different companies in Flanders (Belgium), using a semi-structured questioning route in five themes [personal sitting patterns; intervention strategies during working hours, (lunch) breaks, commuting; and intervention approach]. The audiotaped interviews were verbatim transcribed, followed by a qualitative inductive content analysis in NVivo 10.

**Results:**

The majority of participants recognized they spend their working day mostly sitting and associated this mainly with musculoskeletal health problems. Participants suggested a variety of possible strategies, primarily for working hours (standing during phone calls/meetings, PC reminders, increasing bathroom use by drinking more water, active sitting furniture, standing desks, rearranging the office) and (lunch) breaks (physical activity, movement breaks, standing tables). However, several barriers were reported, including productivity concerns, impracticality, awkwardness of standing, and the habitual nature of sitting. Facilitating factors were raising awareness, providing alternatives for simply standing, making some strategies obligatory and workers taking some personal responsibility.

**Conclusions:**

There are some strategies targeting sedentary time on a working day that are perceived to be realistic and useful. However several barriers emerged, which future trials and practical initiatives should take into account.

## Introduction

Sedentary behaviours -any waking activity characterized by an energy expenditure ≤1.5 metabolic equivalents (METs) while being in a sitting or reclining posture [1,2]- have been identified as a potentially-unique health risk [3,4]. Cross-sectional and longitudinal studies have shown higher amounts of sedentary time, after controlling for moderate-to-vigorous physical activity, to be associated with obesity, metabolic syndrome, type 2 diabetes, cardiovascular disease (CVD), some cancers, mental disorders, and all-cause and CVD mortality in adults [5-16]. Breaks in prolonged sedentary time have been found to be associated beneficially with waist circumference, body mass index (BMI), triglycerides, and 2-hour plasma glucose, independently of the total time spent sedentary [17]. In summary, both the *total amount* of sedentary time and *prolonged periods* of uninterrupted sedentary time may have adverse health effects in adults.

Sedentary time occurs in a variety of settings: the domestic/leisure time environment; the occupational environment; and during transportation, with potentially different behavioural determinants at multiple levels in each of these settings [18]. Occupational sedentary time can be the largest contributor to overall daily sedentary time in white-collar workers [19]. An Australian accelerometer-based study for example revealed that full-time employed adults spent on average 6.8 hours/day sedentary at work [20]. In a review specifically focusing on sedentary time at work [19], the majority of prospective studies found occupational sedentary time to be associated with a higher risk of type 2 diabetes and mortality. Therefore, there is the need to develop and test public health intervention strategies focusing on reducing and interrupting occupational sedentary time. In the process of intervention development [21,22], an important aspect is testing the acceptability (are those likely to be affected by the intervention willing to receive the strategies?) and feasibility (is it realistic to consider implementing the proposed strategies?) of potential intervention strategies in particular contexts. This might prevent implementing an intervention that does not apply to the target population or is for example too costly to implement. A commonly used method to gain detailed insight into what, how and why strategies are found to be acceptable and feasible, is the focus group interview [22].

Only limited evidence is available on acceptable and feasible ways to influence adults’ occupational sedentary time. A qualitative analysis from the USA provided support for the acceptability and feasibility of health-promoting work breaks (Booster Breaks), however barriers were also identified, including a lack of variety in routines and a lack of management support [23]. In an Australian qualitative evaluation of an e-health intervention designed to reduce prolonged occupational sitting, some negative outcomes (disruption of work flow and work habits) were reported, in addition to positive outcomes [24]. Strategies suggested in focus groups with Australian office workers included workload planning (interspersing sitting and non-sitting tasks), environmental changes (e.g. stairwell access, printers away from desks), adding movement to some work tasks (e.g. walking meetings) and purposive physical activity (e.g. periodic breaks, exercise/walking groups) [25]. Enabling strategies identified were team leader involvement and manager support; the main barrier to their implementation was the possibly negative impact on productivity. Another Australian qualitative study with occupational health and safety practitioners revealed ideas for strategies including both offering choices to stand more (e.g. sit-stand desks) and obligating changes (e.g. centralising printers) [26]. Again productivity concerns were cited as a major barrier for change. Qualitative analyses in other (European) countries are recommended in order to generalise findings beyond Australia, [25,26] because workplace management practices, regulations, and cultures can differ between continents or countries [27]. In addition, the concept of sedentary behaviour is very new in Europe and not yet well-known to the general public, which is in contrast to what is the case in other countries, particularly Australia.

In the context of any workplace innovation, executives and senior managers are an important stakeholder group [26] and this is particular the case in relation to understanding the acceptability and feasibility of potential intervention strategies to influence occupational sedentary time. Therefore, qualitative data were collected via focus group interviews of employees’ and executives’ opinions on occupational sedentary time and on potential intervention strategies to reduce and interrupt sedentary time on a working day. The purpose of the present study was to test acceptability and feasibility of developing and implementing workplace interventions to reduce or interrupt sedentary time.

## Methods

### Participants recruitment

One of the researchers (LB) -an occupational physician- identified relevant companies within her network. Selection was based on size (staff n > 100), location (Flanders i.e. the northern, Dutch speaking part of Belgium) and jobs performed in the company (mostly sedentary computer-based tasks). Three companies were randomly selected. The managing boards of these companies agreed to allow their employees and executives participate in this study and provided written informed consent forms. In each company, a contact person was assigned to select a convenience sample of 6–10 employees/executive, [28,29] including both men and women of different ages. No other inclusion or exclusion criteria were used. Details of the focus groups are presented in Table 1.

**Table 1:** **Details of the focus groups and participants**

**Setting**	**Sector**	**Sample size (n)**	**Age (range)**	**Gender (% women)**	**Education (% college/university)**	**Self-reported occupational sitting (mean hours ± SD)**
**Executives**						
Company 1	Secondary/tertiary	5	31-38	40.0%	100.0%	7.3 ± 1.4
Company 2	Secondary	11	30-56	36.4%	100.0%	7.1 ± 0.8
Company 3	Public	5	30-47	60.0%	100.0%	7.7 ± 1.2
**Employees**						
Company 1	Secondary/tertiary	6	23-51	100.0%	50.0%	6.6 ± 1.6
Company 2	Secondary	11	26-64	63.6%	45.5%	6.9 ± 1.1
Company 3a	Public	8	25-59	75.0%	62.5%	7.6 ± 1.7
Company 3b	Public	9	21-52	33.3%	66.6%	7.6 ± 0.4

### Focus group protocol and procedures

In each company, separate focus groups were conducted among employees and executives. The use of this qualitative method in a group format has several advantages, which include gaining relevant underlying insights, perceptions, and assumptions; detecting subtle ambiguities which are hard to assess using quantitative methods; and having a group dynamic which can minimise the impact of extreme views, help to assess the degree to which there is a consistent and shared view, and enable social norms and beliefs to be traced [30]. The protocol was standardised across the different focus groups, however there was flexibility within the procedures to allow for varied question order during the focus groups. This protocol included detailed information on the sampling and the recruitment of participants, the location and settings, timing, recording equipment, guidelines for the moderator and co-moderator to prepare and guide the focus group discussions, and instructions on the data analysis. Prior to the focus group, all participants completed an individual written informed consent form and a two-page questionnaire on demographic variables including age (open-ended), gender, educational level (no diploma/elementary school/secondary school/college/university), and on self-reported sitting time at work (open-ended; based on the Workforce Sitting Questionnaire [31]) (see Table 1).

All focus groups were led by a moderator (CV or KDC) familiar with the questioning route and objectives of the focus groups. A co-moderator (KDC or CV) co-assisted, handled logistics, took notes and orally summarized at the end. The moderator started each focus group with an introduction on the concept and the known health consequences of sedentary behaviour, as well as the framing and guidelines of the focus group.

After each focus group session, the moderator and co-moderator debriefed and discussed the main findings, unexpected outcomes, differences with previous focus groups and global impressions. The focus groups took place at the worksite during working hours or lunch time, lasted up to one hour and all were audiotaped. No incentives were given to participate; however refreshments and biscuits (during working hours) or sandwiches (during lunch time) were provided. This protocol was approved by the Ethical Committee of the Ghent University Hospital.

### Questioning route

A semi-structured questioning route started with general opening questions and several key questions and sub questions. The flexible questioning route was organized in five themes [(1) personal sitting patterns; intervention strategies during (2) working hours, (3) (lunch) breaks, (4) commuting - public transport; (5) overall intervention approach]. All participants commuted either by car or by bike so the theme ‘commuting’ was omitted in the results section as focussing on sedentary behaviour is rather irrelevant in these situations. Sub-questions were used to start or keep the discussion going if this did not happen spontaneously. The questioning routes were similar for employees and executives including personal opinions on their sitting time, and strategies to both reduce or interrupt sitting time. Additionally, employees were asked what the role of the executives should be and executives’ opinions about being the promoter of an intervention were also asked. The order of the questions could be changed depending on how the discussion proceeded.

Table 2 gives an overview of the definitive questioning route. Its original basis was pilot-tested in a convenience sample of research department employees (n = 8), and was adapted where necessary. Adaptations included mention of the independent negative health impact of too much sitting, more focus on the difference between physical inactivity and sitting, and a clear distinction between reducing total sitting time and interrupting prolonged sitting time.

**Table 2:** **Questioning route used in the focus groups among employees and executives: opening and transition questions (regular), key questions (bold) and examples of sub questions (italic)**

	**O** **pening question**
	What is your name and function within the company?
Theme 1	Personal sitting patterns
	When do you usually sit on a regular work day?
	*Do you sit at your desk, during meetings,…?*
	What are the consequences of sitting at work according to you?
	*Do you think these are short- or long-term consequences?*
Theme 2	Intervention strategies during working hours
	**What is your opinion on reducing and interrupting sitting time during work?**
	*Would you be willing to stand up at your desk?*
	**What strategies could be used to reduce or interrupt sitting time at work?**
	*Which possibilities do you see to sit less at your desk?*
	*Which barriers would prevent you from interrupting your sitting?*
Theme 3	Intervention strategies during (lunch) breaks
	Transition: What are your usual routines during (lunch) breaks?
	**What is your opinion on reducing or interrupting sitting time during lunch/other breaks?**
	*Are you prepared to interrupt your sitting time during lunch? Why (not)?*
	**What strategies could be used to reduce or interrupt sitting time during lunch/other breaks?**
	*Which possibilities do you see to achieve a reduction in sitting during lunch?*
	*Which barriers would prevent you from reducing your sitting during lunch time?*
Theme 4	Intervention strategies during commuting - public transport
	Transition: How do you normally commute to and from work?
	**What is your opinion on reducing or interrupting sitting time during public transport?**
	*Would you be willing to stand during public transportation if possible?*
	**What strategies could be used to reduce or interrupt sitting time during public transport?**
	*How would you like to be reminded to interrupt your sitting during public transportation?*
Theme 5	Overall intervention approach
	Transition: Is there a health policy at your organisation and what is the content?
	**What would be a good approach to promote the strategies to influence sitting?**
	*Which factors can facilitate or hinder the promotion of these strategies?*
	**What should be the management’s role in an intervention to influence sitting time at work?** (only for employees)
	*Would the management be prepared to implement an intervention? How can they best act?*
	**How would you feel being the promoter of strategies to influence sitting time at work?** (only for executives)
	*Why would you (not) promote an intervention to influence sitting time at work?*

### Data analysis

First, the audiotaped interviews were verbatim and anonymously transcribed. Second, the available information was analysed and summarized via qualitative data analysis computer software *NVivo 10* (QSR International Pty Ltd. Version 10, 2012). Qualitative content analysis can be defined as “a research method for the subjective interpretation of the content of text data through the systematic classification process of coding and identifying patterns” [32]. Inductive content analysis directly derives coding categories from the raw transcription data [33]. The unit of analysis was defined, as a “segment of text that is comprehensible by itself and contains one idea, episode, or piece of information” [34,35]. In the present study, the unit of analysis included all participants’ opinions and comments on occupational sitting and strategies to reduce or interrupt this. Consequently, all transcripts were repeatedly read to capture the whole and to discover highlighting phrases referring to content areas [36,37]. Mutually exclusive (sub) categories were derived from these grouped content areas, given a first label, checked for overlap, and if necessary revised.

## Results

### Participants

Seven focus groups were conducted within three companies, i.e. a development and testing department of a manufacturer of plastic products (n = 100 staff members); an autonomous municipal port company (n = 160 staff members); a human-resources organisation (n = 305 staff members). Fifty-five full-time employed participants (21 executives), ranging between 21 and 64 years of age, of which half were female (31/55 or 56.4%) participated in the focus groups. The number of participants per focus group ranged from five to eleven, and self-reported occupational sitting time ranged from 6.2 to 7.3 hours/day (see Table 1). Participating employees were administrative clerks of the accountancy, purchasing, personnel, information and communication technology, legal and communication departments. The executives were members of the managing board of these departments.

### Reflections on occupational sitting time

In all focus groups, the majority of employees and executives reported that they believe they spent their working day mostly sitting; and that their work breaks are also mostly spent sitting:

“*I spend a lot of time sitting at my desk*, *also during meetings I sit. I believe I spend more than 90% of my time sedentary”* [male employee]

*“I think I spend at least 7 of the 8 work hours seated”* [female executive]

In addition, nearly all employees and executives reported they believe that diverse health and related complaints, which were mostly musculoskeletal problems including back-, and neck pain are related to sitting; some participants also think concentration problems and fatigue are related to sitting:

*“I have health problems like neck- and back pain … I regularly visit a chiropractor”* [male executive]

*“At the end of the day, I am actually more tired now compared to the time I was teaching and standing more during the day. When I come home now, I have no more energy”* [male employee]

Regarding breaks in prolonged sitting time, many employees and executives believed that they interrupt their sitting regularly, as they mentioned several reasons why they stand up, e.g. coffee, printer, and bathroom visit:

“*I get up a lot: water, printer, toilet, walking over to a colleague…*” [female employee]

*“I never sit more than 30 minutes at my desk without getting up”* [female executive]

However, both employees and executives still had questions regarding the difference between reducing sitting time and interrupting sitting time, and why it is important to reduce and interrupt sitting time:

*“But I am wondering: is it worth it to stand up and to take two steps, is this really better than staying seated?”* [female employee]

*“I don’t know, what is the ratio? How much do you have to move and how long can you sit? It seems like the duration is important”* [female executive]

Also the distinction between being physically active and standing up to interrupt sitting or to replace sitting was not always clear. Additionally, a lot of employees expressed their doubts about the healthiness of prolonged standing.

“*But standing still is not good either, you have to move and be active”* [female employee]

### Acceptability and feasibility of strategies to change occupational sitting

As shown in Table 3, strategies to reduce and interrupt occupational sitting were suggested on the individual, social, organizational and environmental level (see Table 3). In all focus groups, mostly strategies on the social, organizational and environmental level were acceptable, followed by strategies on the individual level. The strategies were perceived as useful, however, for most of the strategies, several barriers were mentioned making the strategies less acceptable and feasible according to this sample.

**Table 3:** **Acceptable strategies to reduce and interrupt sedentary behaviour at work**

**Level**	**Strategies to reduce occupational sitting +** ***quote***	**Strategies to interrupt occupational sitting +** ***quote***
Working hours		
**Individual**	Standing while sorting and filing paper reports when there is no digital database	Computer reminders
		*“A computer reminder would make one more aware: ‘you are already being seated for 4 hours and a half’, the system says… even if it is only making us conscious, that’s a lot”* [male employee]
		Active sitting furniture
		*“I think it’s a good idea to sit actively on a gym ball, as in this way the step to standing up is smaller compared to when you are sitting comfortably in a chair”* [female employee]
		Drink more water and go to the toilet more often
**Social**	Walking or biking conversations	Walking over to a colleague
	*“This is indeed something positive. Besides being active, the conversation may be even smoother, that’s a win-win”* [female employee]	*“This is only a small effort that breaks your sitting, plus it improves the contact with the colleague”* [male employee]
**Organizational**	Standing meetings (max 15 minutes) in small groups	Standing breaks in middle of meeting or after 30 minutes
	*“The more you sit, the longer you discuss, the longer the meeting lasts, so…standing may be more efficient”* [female employee]	*“Wouldn’t it be good to decide to have a standing break after 30 minutes when meetings will last more than one hour?”* [female employee]
	Those who speak stand up (only for executives)	
	“*You could also agree that the one who is talking should stand up during the meeting. This is a small effort. If we could get this mentality, it’s not much effort and feasible for everyone”* [male executive]	
**Environmental**	Adjustable standing desks (individual use)	Central printers/bins/coffee corners/water stations/mail boxes
	*“We should have a desk that can move up, it may sound silly but then I would stand up for one hour”* [male employee]	*“I think we can achieve a lot by putting the printers and coffee machines further away”* [female executive]
	Separate meeting room with standing tables + extra trigger to use that room	
	*“Why should you use that room (= room with standing tables) if there is an alternative where you can sit, I believe an extrinsic motivation is needed, like fruit”* [male employee]	
Lunch time and other breaks		
**Individual**	Active lunch breaks: individual or common	Individual ‘movement break’
	*“During lunch I would suggest to walk outside instead of putting people around a table again”* [male employee]	*“If I for example take a break for 5 minutes every two hours, I think I will restart work with double the amount of concentration, so I believe we have benefits for our body but also for the concentration”* [male employee]
**Organizational/**		
**Environmental**	Standing tables in canteens for cold lunch	
	*“It’s okay to stand while eating sandwiches, but if you order a soup or a hot meal, this (=standing) is really not comfortable and really not practical…”* [female executive]	

First, the fear of being (seen as) unproductive or getting other negative reactions was pointed out as a general barrier. A lot of employees had the feeling that executives and colleagues would not appreciate it when employees are not sitting at their desk and working, however executives themselves didn’t seem to have a problem with this.

*“I have a colleague who drinks water out of little cups instead of a bottle, so every time his cup is empty he gets up to refill it, so he walks around the whole time with the consequence that people start thinking ‘he has nothing to do, he is constantly walking around’”* [female employee]

*“I wouldn’t have that reaction (*i.e.*, someone is walking around so he/she is being unproductive) at all but I believe that others would think like that”* [female executive]

In addition, some strategies were considered impractical, such as standing during phone calls when computers or documents are needed, or having standing meetings when notes should be taken:

*“You really need higher tables to take notes or to take a look at your papers, you need to have these facilities otherwise people will sit if they have the opportunity”* [female executive]

*“Interrupting meetings to stand up is really like a ‘break’ in your meeting. When you are discussing things and suddenly saying ‘let’s stand up’… I don’t know if this is always feasible” [female employee]*

Further, financial aspects were found to be a barrier for the use of standing desks, which was however perceived as an acceptable strategy:

*“I think it (*i.e.*, standing desks) is original, but in terms of feasibility? It is a serious cost investment in furniture of course*” [male employee]

The fact that standing could or would disturb others was also mentioned as a barrier:

*“When you call wireless you can stand or walk while being on the phone but then you have the risk of disturbing others (laughs)”* [female executive]

In addition, standing was perceived as awkward in certain situations:

*“I would feel like being in church when standing up at a certain point”* [male employee]

*“Not everyone is enthusiastic or comfortable to stand up while speaking, I think it will block certain people more than it will motivate them”* [female employee]

Finally, the habitual nature of sitting was mentioned as barrier to change occupational sitting:

*“Once you are seated, you just stay seated”* [male employee]

*“It’s like with ‘solo desks’ which are telephone-free, the threshold is too high to use it, people don’t easily leave their usual work site”* [female executive]

### Intervention delivery, content and implementation

In all focus groups, the following ways were mentioned to inform people and to communicate about intervention strategies: intranet, personal contact, posters, and/or the Internet.

Both employees and executives expressed beliefs that a component of any intervention should be raising awareness and providing information:

*“I think it would be interesting to point out the negative consequences (of sitting) as everyone thinks that sitting in a chair is only bad for their backs”* [male employee]

*“For example a measuring campaign can help to confront people with how long they really sit…people may react like ‘Oh, I just stayed seated for two hours without any movement’!”* [male executive]

Another perceived overall facilitating factor was ‘providing a reason or giving an alternative for simply standing’:

*“It will depend on how the message will be brought, if we can motivate and point out the usefulness, I think it’s feasible, but I don’t see any advantage in just implementing things without having a good reason”* [female executive]

Some suggested that the initiatives should be short and change regularly or be competitive:

*“There are a lot of (health) initiatives, but if they last too long, they will die silently, therefore I prefer short initiatives that change now and then”* [female executive]

*“Yes, a game or a competition to motivate people… I think this is needed to get something off the ground”* [male executive]

Both employees and executives also reported that a change in workplace culture would be beneficial to achieve behavioural change:

*“We should create a more healthy climate in the office… I once suggested to have fruit plates and someone laughed ‘one piece of fruit won’t make a difference’. I know that, but the culture can make a difference! The overall picture should be right, people here sit the whole day, eat unhealthy, drink coffee the whole day…!”* [female employee]

*“I don’t know if we have the ability to follow the example of Japan, they interrupt their work to do some exercises, but I think if you implement this here (laughing)… the time is not right for this”* [male executive]

There was disagreement on how to best implement strategies. Executives mostly believed that people should make their own choices on how and when to reduce and/or interrupt sitting time (for example, standing tables should be offered next to regular tables, only providing standing tables or obligating people to use the standing tables was not seen to be an option):

*“I think the solution is to provide a range of possibilities to the employees and let them be responsible for their own actions”* [male executive]

Instead most employees preferred to make some strategies mandatory:

*“There’s a difference between people who take partial responsibility to change and people who need to be required to do something. I believe there are people within this company who like to be obligated and wait for others to take an initiative”* [male executive]

*“I think that when it is described in the job task to stand up, one will do this. I believe it is the only solution to make one standing up during the job, otherwise it won’t happen”* [male employee]

The majority of employees reported that they would appreciate executives who act as a role model or take the first step in implementing strategies:

*“I think it’s positive if the initiative is a top down approach”* [male employee]

*“In fact, executives should support this and motivate us to stand up”* [female employee]

Executives were willing to take this role; however they believed the employees have a responsibility as well:

“*I think it’s a bit strange that employees need us to become active or less sedentary; however we can act like a model* …” [male executive]

*“I believe there are other trendsetters and motivators besides the executives*” [female executive].

## Discussion

Focus group interviews with Flemish employees and executives identified several relevant sets of perceptions of occupational sitting and the acceptability and feasibility of workplace intervention strategies to reduce and interrupt occupational sitting. These findings have implications in European context for the development and implementation of interventions aimed at influencing sitting at work.

These employee and executive reflections on occupational sitting point out the potential lack of knowledge concerning the current evidence on sedentary behaviour, resulting in several questions about health-related sedentary behaviour (how long can we sit?, why should we interrupt?), doubts about the healthiness of standing, and linking sitting particularly with musculoskeletal health problems. The latter was also found in another study [25]. The European tradition of occupational health and safety regulations strongly focussing on ergonomics may be an explanation for the present way of thinking of employed adults [38,39]. The findings also point out that in Europe people are not familiar with the concept of sedentary behaviour and health and do not yet see this behaviour independent of physical inactivity, which is in contrast with Australian findings [25]. These results imply that more information or education is needed on the recent insights regarding sedentary behaviour, including the independent metabolic and chronic health risks of prolonged sitting. In previous worksite interventions promoting health-related behaviours other than sitting, (group) education was also part of the programs [40,41] and a review showed that the provision of education was an effective strategy to promote physical activity in the workplace [42]. Future studies will need to confirm the impact of educational aspects in workplace interventions aimed at influencing sitting, however a pre-intervention education session was found to be an effective mechanism for decreasing prolonged employees sedentary periods and increasing movement throughout the workday in Australian desk-based workers [43]. In practice, it might be convenient to add the current sedentary behaviour knowledge to existing ergonomic programs and to sensitize occupational physicians and workplace prevention coordinators to support this evidence. Present results also plead for the development of clear evidence-based health-related guidelines of (domain-specific) sedentary behaviour, next to those for physical activity [44,45], as these are not available in the literature at this moment.

The strategies identified for reducing and/or interrupting occupational sitting time correspond to the ecological model on sedentary behaviour, as strategies at the individual, social, organizational and environmental level were suggested [18]. Overall, the strategies found to be acceptable were mainly similar to those suggested in focus groups among Australian employees [25] and those implemented in a multicomponent intervention in Australian office workers [46]. Further research is needed to examine the actual influence of these suggested strategies on behaviour change in future interventions focussing on occupational sitting; however the evidence of effectiveness at this stage, while limited, is nevertheless promising [46,47].

The main barriers seen to be compromising the acceptability and feasibility of the strategies were comparable to those found in similar studies. In the focus groups of Gilson et al. [25], the potential loss of productivity and focus was the primary barrier for employees, followed by negative responses from management. Australian occupational health and safety (OHS) practitioners also had productivity concerns as a main influence for change [26] and a negative outcome reported in the evaluation of a workplace intervention to reduce prolonged occupational sitting was the disruption of the work flow [24].

Concerns about the practicality, cost and organization of several strategies were also reported in the focus groups of Gilson et al. [25]. The use of standing desks for example, implies a substantial perceived investment cost making this acceptable strategy less feasible. A qualitative evaluation of sit-stand desks in an Australian workplace showed, however, that sit-stand desks had high usability and acceptability and reduced sitting time at work [48].

The present findings have some practical implications for future interventions:

First, given the fact that sitting was recognized to be an easy habit which is hard to unlearn, continuous awareness raising seems needed. A study of Conroy et al. [49] indeed confirms that sitting is a daily process partly regulated by habits.

Second, simply asking people to stand up was perceived not to be acceptable by the study participants, suggesting that employees currently are not being autonomously motivated to change their sitting and stand up [50,51]. Alternatives to only standing as an alternative to sitting seem to be needed in order to promote change in sitting behaviour; and potentially a motivational intervention component might be needed to assist workers to formulate their own relevant alternative strategies.

Third, strategies should be simple and easy to implement, while not decreasing productivity and task focus. Employees’ perceptions of social support from management should be increased, which may happen if executives act as role models. The latter was also reported as an enabling factor by the present employees. In a study evaluating health promoting work breaks, a need for greater management support was also identified [23]. Having role models in the media, together with more media attention to workplace sitting, may also help to increase the perception of social support.

Finally, there is a need to take into account what has emerged from our study showing that employees and executives can think differently about some of the factors related to workplace sitting. Multiple approaches may be needed. For example, making some strategies obligatory was considered to be a facilitating factor for employees, while executives believed that employees should have the personal choice to decide about such matters for themselves. Such an apparent disjunction between providing employees with personal choices and making sitting-related changes obligatory was also identified from focus groups conducted with Australian OHS practitioners [26]. The fact that the participants in our study identified sitting as a strong habit that can be difficult to change may be related to employees identifying that some forms of organisational compulsion may be required. However, any suggestions about the use of a forced computer lock-out system were rejected immediately during these focus groups, while there were more positive reactions identified in Australian focus groups [25]. The present finding is also in contrast to what emerged among Australian employees of a professional workplace who were willing to accept a 26 weeks persuasive e-health intervention system with an element of compulsion [52]. Moreover, in this Australian study, the pop-up prompt, deactivating the screens every 45 minutes, was found to be effective in increasing non-purposeful movement at work [53]. Another aspect on which employees and executives did not fully agree was the intervention approach. Employees expected a top down intervention (with some obligatory strategies), while executives believed that intervening should be a shared responsibility. In Australian employees, workplace interventions were considered to be a joint responsibility of employees and the organisation [25].

The present study has some limitations that need to be considered. First, participation was voluntary, which may have resulted in recruiting employees and executives who potentially have unrepresentative perspectives. In addition, the sample of employees was mainly female. These weaknesses may compromise representativeness of the sample and generalizability of the findings. Furthermore, because of the open-ended recruitment approach that was used, information is not available on how many of those potentially eligible did not express interest in participating. Second, it cannot be assumed that participants have a comprehensive understanding and insight into the nature and extent of their own occupational sitting, nor of the causes and health consequences of sitting too much. These findings are perceptions of the participants and should not be interpreted as representing what is actually the case in their workplaces. However, we believe that these perceptions are of relevance and can be taken into account when developing intervention strategies to address occupational sitting. Finally, no participants used public transport to commute, resulting in the fact that this theme was not addressed during the focus groups. A unique aspect of our study is that our focus groups included executives, which is one of the major strengths of the present study. In addition, no other European qualitative study on this topic has yet been reported.

In summary, the present findings indicate that Flemish employees and executives were largely unaware of current knowledge about sedentary behaviour and health and the implications of this evidence for workplace policy and practice. Nevertheless, potentially relevant and plausible strategies mainly at the social, and environmental level were suggested to primarily interrupt sitting time during working hours. Overall, such strategies were perceived as potentially useful. However, the implementation of these strategies during working hours appeared to our participants to be likely to be unacceptable or unfeasible, as most strategies were perceived to be counteracted by barriers, such as concerns about productivity, practicality and costs, the awkwardness of standing, and the habitual nature of sitting. Overall, raising awareness, providing health-related alternatives for standing, providing some acceptable obligating strategies for employees, but also leaving the choice to decide and employees taking responsibility were considered to be facilitating factors and would increase acceptability of the intervention.

## Conclusions

This study provides new perspectives with the potential to inform the development of workplace sitting interventions in a European context. However, additional research into the factors influencing occupational sitting time in different countries and in the different context of their workplace cultures and practices, is needed. At this stage, it seems likely that future programs need to be multi-component interventions focusing on informational, educational and motivational strategies on the individual, social, organizational, and environmental level. The effectiveness and sustainability of these strategies should be examined in further research.

## Abbreviations

MET: Metabolic equivalent
CVD: Cardiovascular disease
BMI: Body mass index
OHS: Occupational health and safety
